# Bis(mefloquinium) butane­dioate ethanol monosolvate: crystal structure and Hirshfeld surface analysis

**DOI:** 10.1107/S2056989019009654

**Published:** 2019-07-12

**Authors:** James L. Wardell, Mukesh M. Jotani, Edward R. T. Tiekink

**Affiliations:** a Fundação Oswaldo Cruz, Instituto de Tecnologia em Fármacos-Far Manguinhos, 21041-250 Rio de Janeiro, RJ, Brazil; b Department of Physics, Bhavan’s Sheth R. A. College of Science, Ahmedabad, Gujarat 380001, India; c Research Centre for Crystalline Materials, School of Science and Technology, Sunway University, 47500 Bandar Sunway, Selangor Darul Ehsan, Malaysia

**Keywords:** crystal structure, mefloquine, salt, hydrogen bonding, Hirshfeld surface analysis

## Abstract

The asymmetric unit of the centrosymmetric title salt solvate, 2C_17_H_17_F_6_N_2_O^+^· C_4_H_4_O_4_^2−^·CH_3_CH_2_OH, (systematic name: 2-{[2,8-bis­(tri­fluoro­meth­yl)quinolin-4-yl](hy­droxy)meth­yl}piperidin-1-ium butane­dioate ethanol monosolvate) comprises two independent cations, with almost superimposable conformations and each approximating the shape of the letter *L*, a butane­dioate dianion with an all-*trans* conformation and an ethanol solvent mol­ecule. In the crystal, supra­molecular chains along the *a*-axis direction are sustained by charge-assisted hy­droxy-O—H⋯O(carboxyl­ate) and ammonium-N—H⋯O(carboxyl­ate) hydrogen bonds. These are connected into a layer *via* C—F⋯π(pyrid­yl) contacts and π–π stacking inter­actions between quinolinyl-C_6_ and –NC_5_ rings of the independent cations of the asymmetric unit [inter-centroid separations = 3.6784 (17) and 3.6866 (17) Å]. Layers stack along the *c*-axis direction with no directional inter­actions between them. The analysis of the calculated Hirshfeld surface reveals the significance of the fluorine atoms in surface contacts. Thus, by far the greatest contribution to the surface contacts, *i.e.* 41.2%, are of the type F⋯H/H⋯F and many of these occur in the inter-layer region. However, these contacts occur at separations beyond the sum of the van der Waals radii for these atoms. It is noted that H⋯H contacts contribute 29.8% to the overall surface, with smaller contributions from O⋯H/H⋯O (14.0%) and F⋯F (5.7%) contacts.

## Abstract 2

As the piperidin-1-ium group is nearly orthogonal to the quinolinyl residue in each of the two independent cations of the title salt solvate, these cations are l-shaped. Supra­molecular chains arise in the crystal as a result of charge-assisted O—H⋯O and N—H⋯O hydrogen bonding.

## Chemical context   

Malaria continues to be a major worldwide health issue and vast populations in tropical countries, including visitors to those regions, are susceptible to the disease, which is spread by parasites such as *Plasmodium falciparum* (Maguire *et al.*, 2006 ▸). The problem is compounded by the parasites’ abilities to develop resistance to drugs, such as to the once popular chloro­quine (Grabias & Kumar, 2016 ▸). Mefloquine, [2,8-bis(tri­fluoro­meth­yl)quinolin-4-yl]-piperidin-2-yl­methanol, is a drug used against malaria (Tickell-Pa­inter *et al.*, 2017 ▸). The mol­ecule contains two adjacent chiral centres, *i.e*. one at the carbon atom carrying the hy­droxy group and one at the link connecting the piperidinyl ring to the rest of the mol­ecule. The drug is commonly marketed as *Lariam*, which is the hydro­chloride salt, comprising (R*,S*)-(2-{[2,8-bis­(tri­fluoro­meth­yl)quinolin-4-yl](hy­droxy­meth­yl)piperidin-1-ium chloride and its (S*,R*) enanti­omer. While the former is effective against malaria, the latter has an affinity for the adenosine acceptors in the brain, inducing serious psychiatric and neurologic side-effects (Nevin, 2017 ▸). Hence, experiments at resolving the enanti­omers are of practical importance (Engwerda *et al.*, 2019 ▸). Herein, as continuation of our anion-exchange experiments of the racemic salt and attendant structural studies (Wardell *et al.*, 2016 ▸; Wardell, Wardell *et al.*, 2018 ▸; Wardell, Jotani *et al.*, 2018 ▸; Wardell & Tiekink, 2019 ▸), the crystal and mol­ecular structures of the butane­dioate salt, isolated as an ethanol monosolvate, are described along with an analysis of the calculated Hirshfeld surfaces.
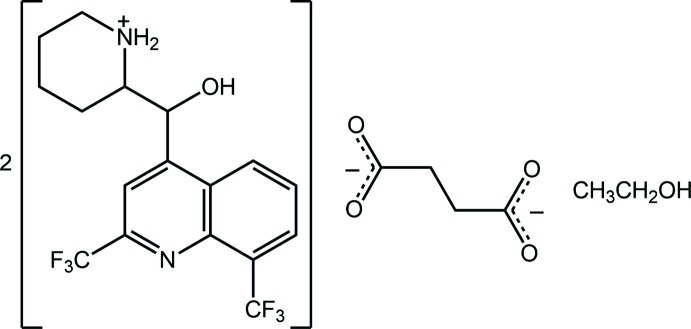


## Structural commentary   

**Figure 1:**
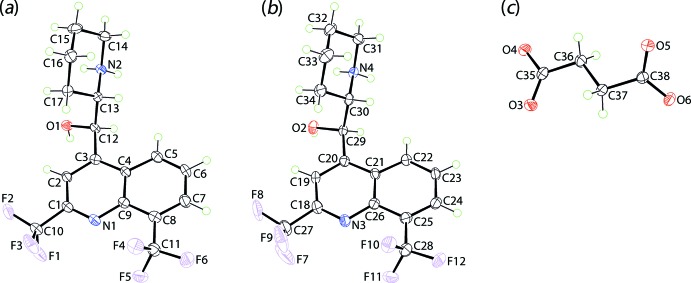
The mol­ecular structures of the ionic components of the asymmetric unit of (I) showing the atom-labelling scheme and displacement ellipsoids at the 50% probability level: (*a*) the N1-containing cation, (*b*) the N3-cation and (*c*) the butane­dioate dianion.

The asymmetric unit of the salt solvate, (I), comprises two mefloquinium cations, a butane­dioate dianion and a solvent ethanol mol­ecule; the mol­ecular structures of the ions are shown in Fig. 1 ▸. Evidence of proton transfer during crystallization is seen in the relatively small difference in the C O bond lengths of the dianion, *i.e*. C35—O3, O4 = 1.236 (4) and 1.285 (3) Å, and C38—O5, O6 = 1.255 (4) and 1.271 (4) Å. While normally these bond lengths might be expected to be closer to equivalent, as noted below, each of the O4 and O6 atoms participate in two strong charge-assisted hydrogen bonds, see *Supra­molecular features*, which explains the slightly longer C O bond lengths formed by these atoms. Further support for proton transfer leading to the formation of piperidin-1-ium cations is supported by the pattern of hydrogen bonding involving the ammonium-N—H hydrogen atoms, as discussed below in *Supra­molecular features*.

**Figure 2:**
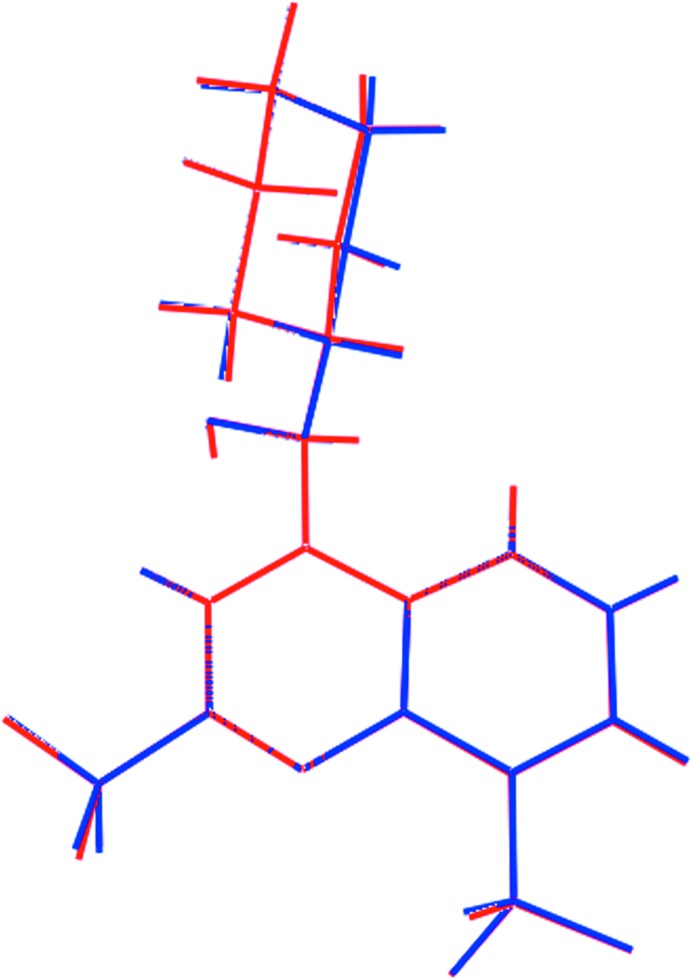
An overlay diagram of the N1- (red image) and N3-containing cations. The cations have been superimposed so that the C_5_N rings of the quinolinyl residues are coincident.

The cations exhibit very similar mol­ecular geometries, as highlighted in the overlay diagram of Fig. 2 ▸. There are two chiral centres in each cation and the illustrated cations are *R* at C12 and *S* at C13 for the N1-cation, and *R* at C29 and *S* at C30 for the N3-cation, *i.e*. each conforms to the [(+)-*erythro*-mefloquinium] isomer; space-group symmetry indicates that the unit cell contains equal numbers of both enanti­omers. The r.m.s. deviation for the ten atoms comprising the N1-quinolinyl residue is 0.0254 Å [0.0256 Å for the N3-quinolinyl residue], with the hy­droxy-O1 and ammonium-N2 atoms lying to either side of the plane, *i.e*. −0.323 (4) and 1.302 (6) Å, respectively [0.255 (4) Å for O2 and −1.348 (6) Å for N4]. The dihedral angle of 72.55 (9)° [71.48 (9)°] formed between the fused ring system and the least-squares plane through the piperinium ring indicates that, to a first approximation, the mol­ecule has the shape of the letter *L*. Referring to Table 1 ▸, an intra­molecular charge-assisted ammonium-N^+^—H⋯O(hy­droxy) hydrogen-bond is formed as the hydroxyl-O1 and ammonium-N2 atoms lie to the same side of the cation with the O1—C12—C13—N2 torsion angle of −63.4 (3)° indicating a + syn-clinal relationship [O2—C29—C30—N4 = −68.4 (3)°].

In the butane­dioate dianion, the C35—C36—C38—C39 torsion angle of 175.4 (3)° indicates an all-*trans* conformation (+ anti-periplanar). The dihedral angle formed between the terminal carboxyl­ate residues is 51.0 (2)°, indicating that the dianion is considerably twisted.

## Supra­molecular features   

**Figure 3:**
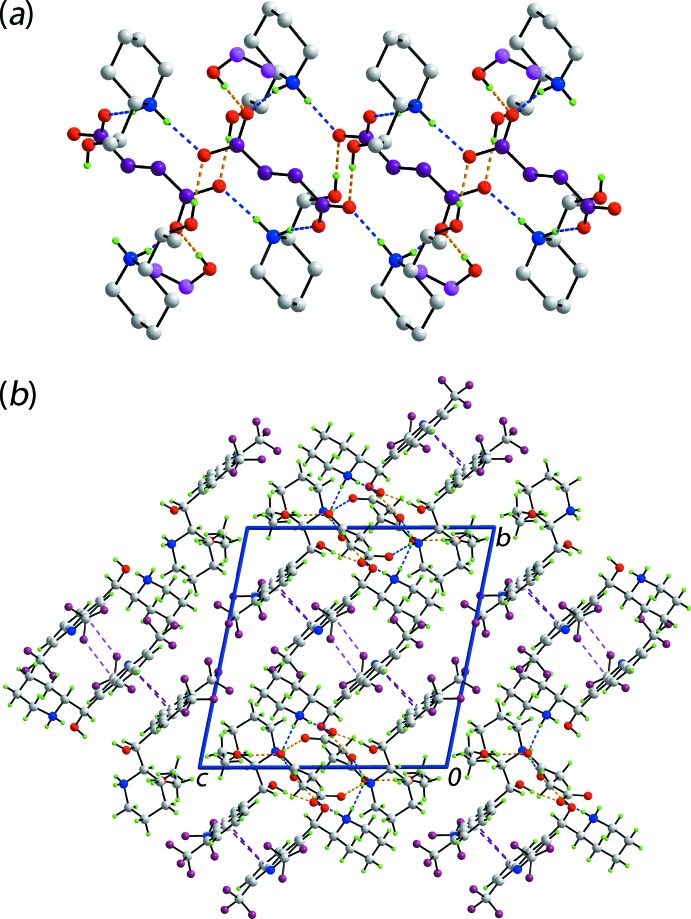
Mol­ecular packing in (I): (*a*) The supra­molecular chain along the *a* axis, being sustained by O—H⋯O (orange dashed lines) and N—H⋯O (blue dashed lines) hydrogen bonding with non-participating H atoms omitted and (*b*) a view of the unit-cell contents shown in projection down the *a* axis, the axis of propagation of the chain shown in (*a*). The C—Cl⋯π, and C—F⋯π inter­actions are shown as pink and purple dashed lines, respectively.

The most prominent feature of the mol­ecular packing is the formation of twisted supra­molecular chains propagating parallel to the *a*-axis direction, Table 1 ▸ and Fig. 3 ▸ *a*. Each of the cation-bound hy­droxy groups forms a charge-assisted hy­droxy-O—H⋯O(carboxyl­ate) hydrogen bond to a carboxyl­ate-O atom, at opposite ends of the butane­dioate dianion. In addition, each of the four ammonium-N—H hydrogen atoms connects to a carboxyl­ate-O atom, each derived from a different carboxyl­ate residue, *via* a charge-assisted ammonium-N—H⋯O(carboxyl­ate) hydrogen bond. Thus, each of the O4 and O6 atoms accept two charge-assisted hydrogen bonds. The carboxyl­ate-O5 atom accepts a hydrogen bond from the solvent ethanol mol­ecule, while ethanol-O7 participates in a methine-C—H⋯O inter­action, Table 1 ▸. The carboxyl­ate-O3 atom forms only one hydrogen bond. The number and strength of hydrogen bonds formed by the carboxyl­ate-O atoms correlates with the magnitude of the C O bond lengths, *e.g*. the C35—O3 < C38—O5 < C38—O6 C35—O4 (see comment in *Structural Commentary*).

The connections between the chains leading to supra­molecular layers that stack along the *c*-axis direction are of the type C—F⋯π(pyrid­yl), Table 1 ▸, occurring between N1-containing cations, and π–π stacking inter­actions between the independent mol­ecules comprising the asymmetric unit. The latter occur between the C_6_ ring of the N1-quinolinyl residue (C4–C9) and each of the N3-quinolinyl-bound pyridyl (N3,C18–C21,C26) [inter-centroid separation = 3.6784 (17) Å, angle of inclination = 4.27 (14)°] and C_6_ (C21–C26) [3.6866 (17) Å, angle of inclination = 3.67 (14)°] rings. A view of the unit-cell contents is shown in Fig. 3 ▸ *b*.

## Hirshfeld surface analysis   

The analysis of Hirshfeld surface calculations for (I) was performed in order to learn more about the supra­molecular association, in particular, about the inter-layer connections, following established procedures (Tan *et al.*, 2019 ▸) and employing *Crystal Explorer 17* (Turner *et al.*, 2017 ▸). Such analyses have proven useful for salts with multiple components comprising the asymmetric unit (Jotani *et al.*, 2019 ▸).

**Figure 4:**
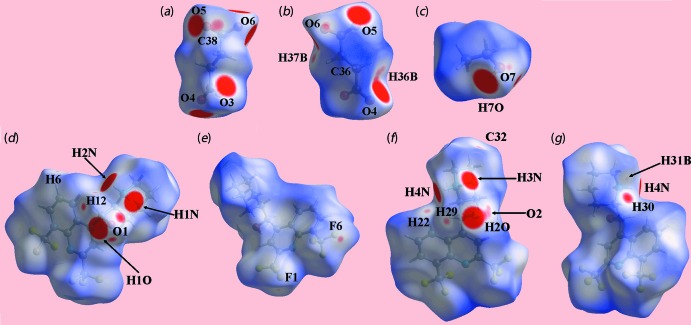
Views of the Hirshfeld surface of (I) mapped over *d*_norm_ for the: (*a*) and (*b*) dianion in the range −0.229 to + 1.450 arbitrary units (a.u.), (*c*) ethanol mol­ecule (−0.169 to +1.471 a.u.), (*d*) and (*e*) O1-containing cation (−0.229 to + 2.242 a.u.) and (*f*) and (*g*) O2-containing cation (−0.219 to +2.159 a.u.).

**Figure 5:**
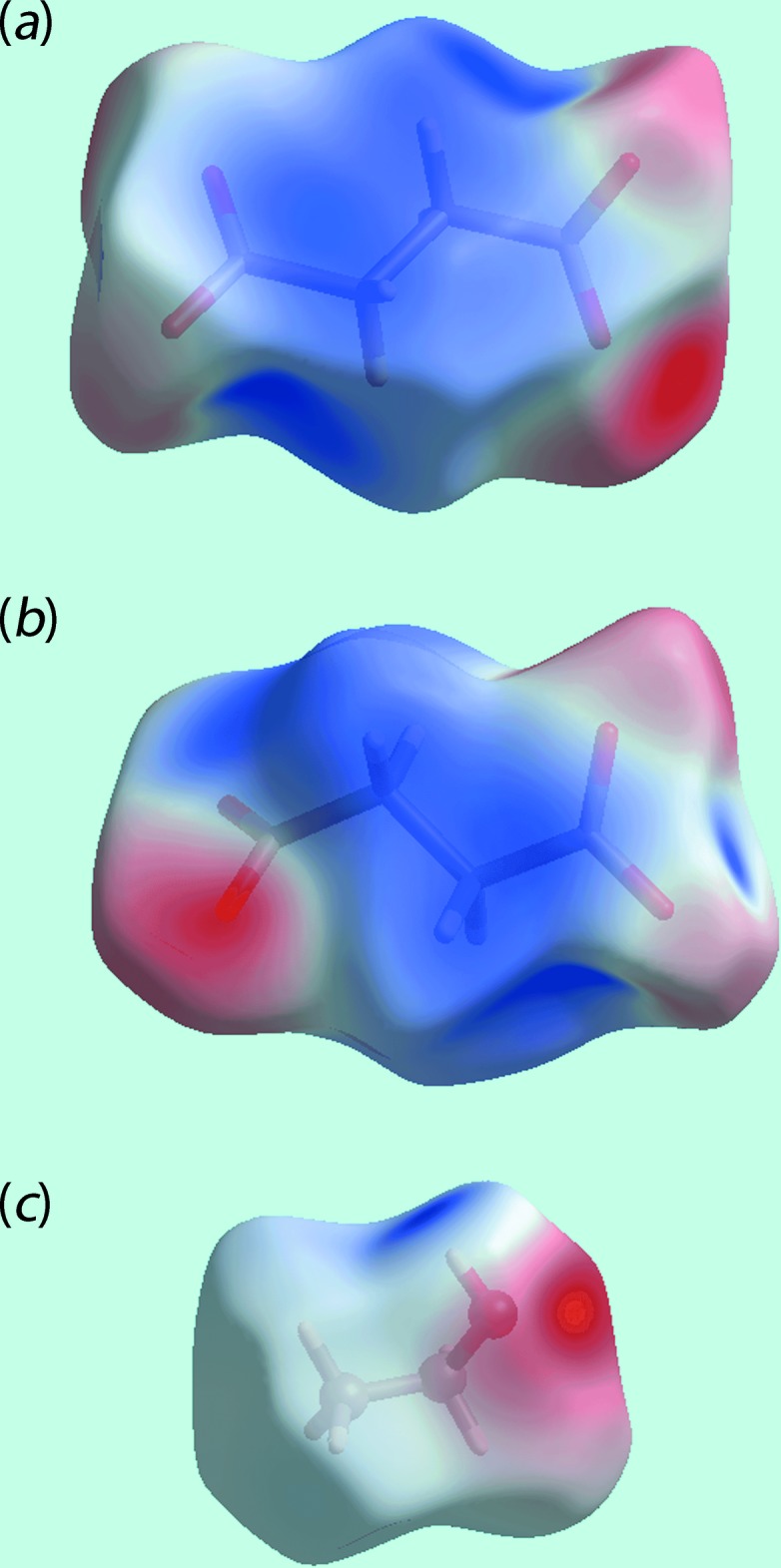
Views of Hirshfeld surface mapped over electrostatic potential for the: (*a*) and (*b*) dianion in the range −0.072 to +0.066 atomic units (au) and (*c*) ethanol mol­ecule (−0.077 to +0.159 au). The red and blue regions represent negative and positive electrostatic potentials, respectively.

**Figure 6:**
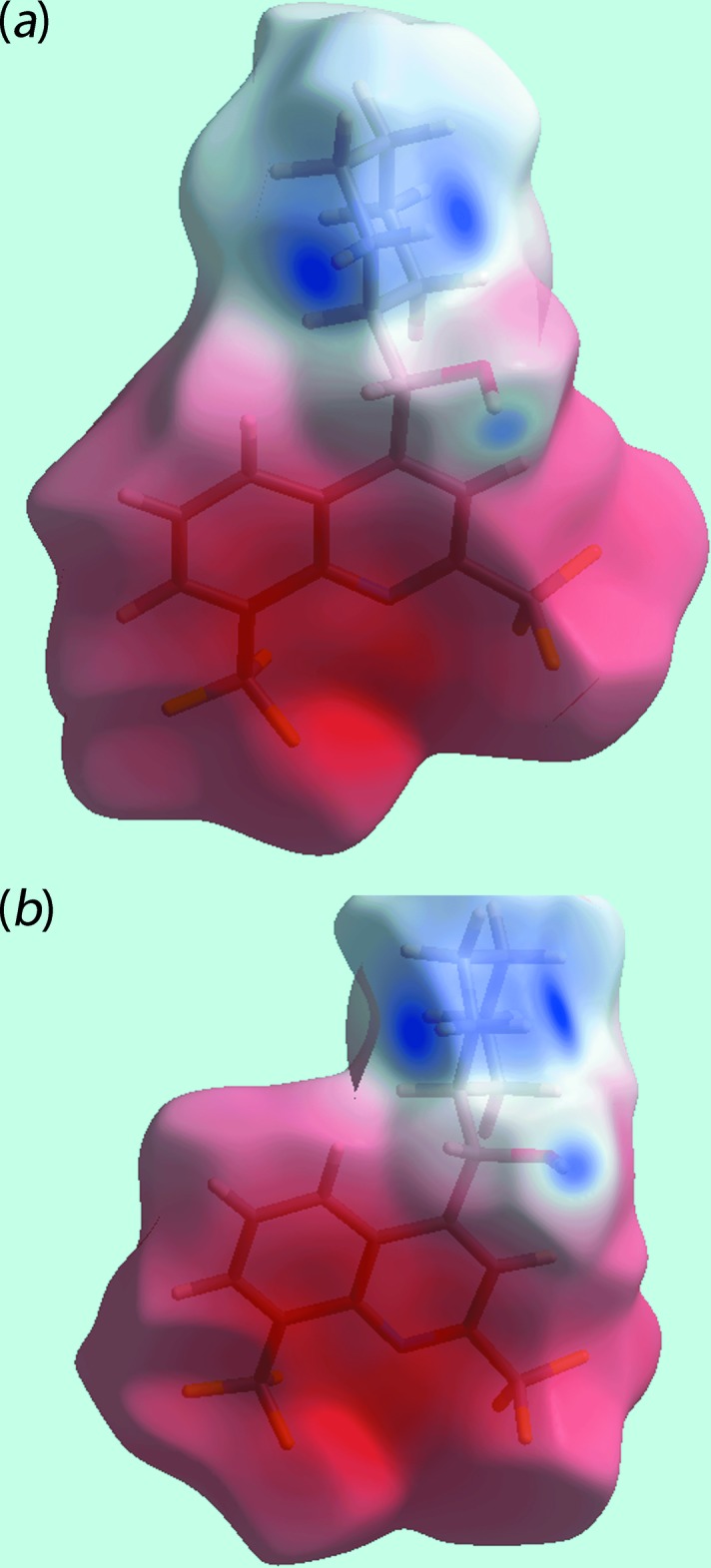
Views of Hirshfeld surface mapped over electrostatic potential for the: (*a*) O1-containing cation in the range −0.262 to +0.215 atomic units (au) and (*b*) O2-containing cation (−0.255 to +0.198 au). The red and blue regions represent negative and positive electrostatic potentials, respectively.

**Figure 7:**
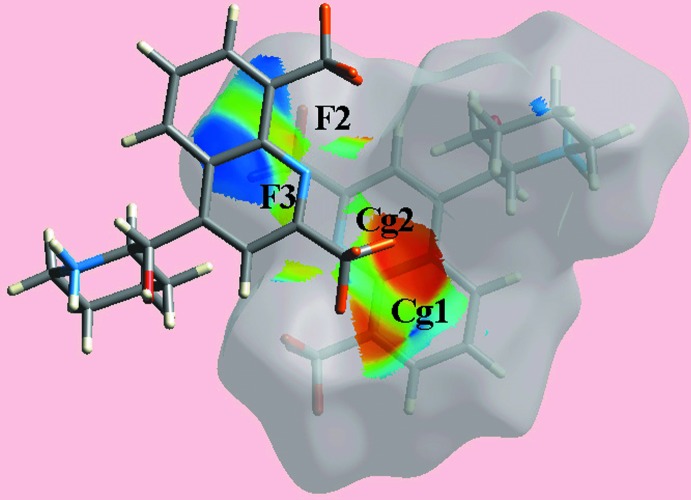
A view of Hirshfeld surface mapped over the shape-index property highlighting the inter­molecular C—F⋯π/π⋯F—C contacts by blue bumps and bright-orange concave regions.

**Figure 8:**
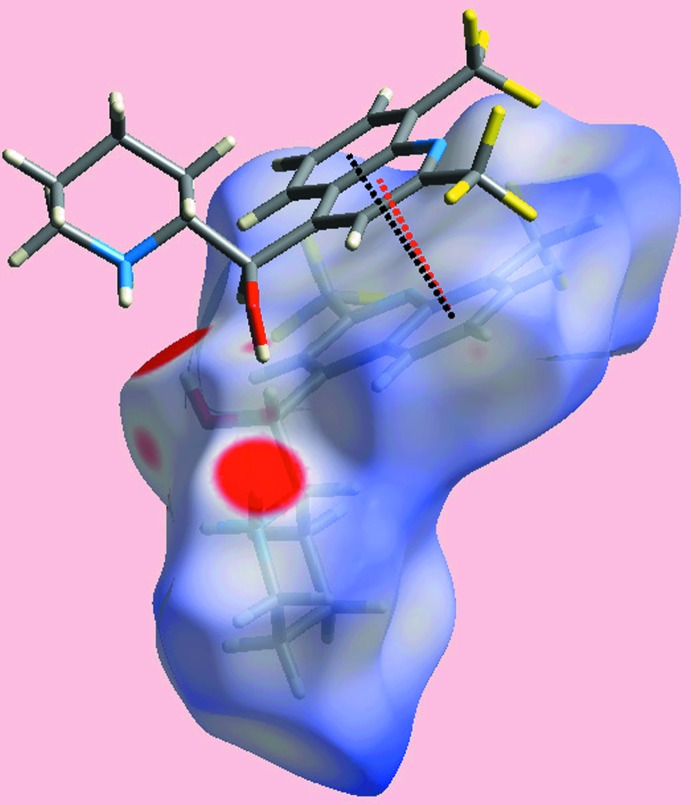
A view of Hirshfeld surface mapped over *d*_norm_ for the O1-containing cation in the range −0.229 to + 2.242 arbitrary units highlighting the intra­molecular π–π contacts between the (C4–C9) ring of the O1-containing cation and the (C21–C26) and N3-pyridyl rings of the O2-containing cation by red and black dotted lines, respectively.

It is clearly evident from the numerous characteristic red spots on the Hirshfeld surfaces mapped over *d*_norm_ for the constituents of (I), shown in Fig. 4 ▸, that the butane­dioate dianion plays a crucial role in forming significant inter­actions with each of the two independent mefloquinium cations as well as with the ethanol solvent mol­ecule. The O—H⋯O and N—H⋯O hydrogen bonds summarized in Table 1 ▸ are characterized as bright-red spots on the Hirshfeld surface mapped over *d*_norm_ for the dianion, Fig. 4 ▸ *a* and *b*, and near the respective donors on the Hirshfeld surfaces of the ethanol mol­ecule, Fig. 4 ▸ *c*, and mefloquinium cations in Fig. 4 ▸ *d* and *e*. The effects of the short inter-atomic contacts on the packing of (I), summarized in Table 2 ▸, are also evident as the faint-red spots near the respective atoms, Fig. 4 ▸. The blue and red regions corresponding to positive and negative potentials, respectively, around the atoms of the dianion and solvent ethanol mol­ecule, Fig. 5 ▸, and cations, Fig. 6 ▸, on the Hirshfeld surfaces mapped over electrostatic potential also represent donors and acceptors of the respective hydrogen bonds. The additional influence of the C—F⋯π contacts involving the F2 and F3 atoms inter­acting with the (C4–C9) and N1-pyridyl rings of the N1-quinolinyl residue are viewed as blue bumps and bright-orange concave regions, respectively, on the Hirshfeld surface mapped with the shape-index property in Fig. 7 ▸. The π–π contacts formed between the (C4–C9) ring of the O1-cation and each of the (C21–C26) and N3-pyridyl rings of the O2-cation are illustrated in Fig. 8 ▸.

**Figure 9:**
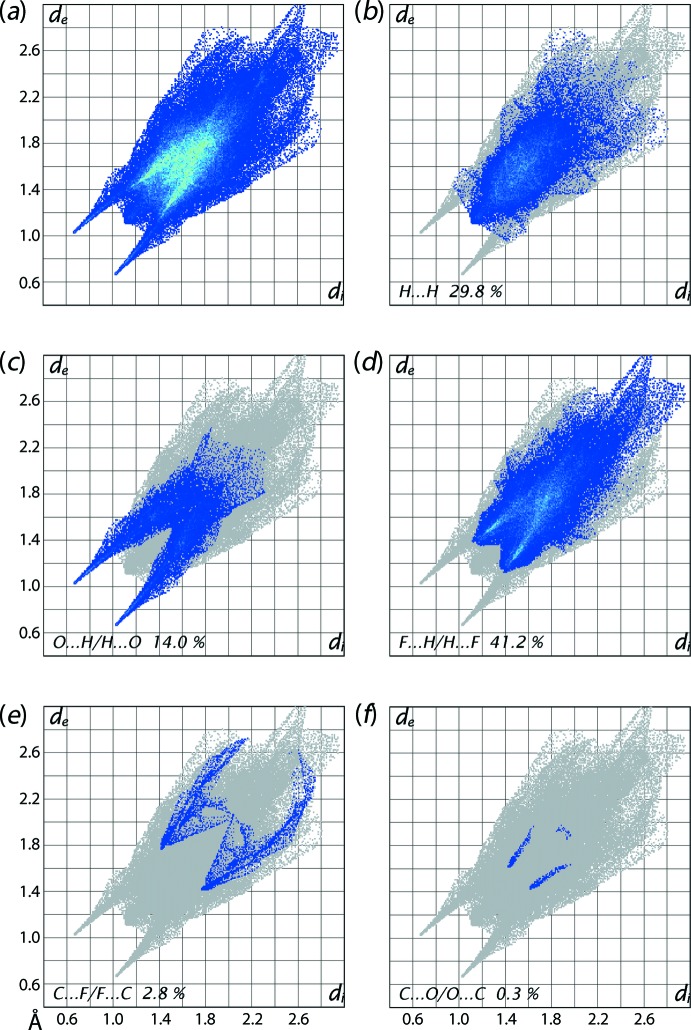
(*a*) The full two-dimensional fingerprint plot for (I) and (*b*)–(*f*) those delineated into H⋯H, O⋯H/H⋯O, F⋯H/H⋯F, C⋯F/F⋯C and C⋯O/O⋯C contacts, respectively.

The overall two-dimensional fingerprint plot for (I), Fig. 9 ▸ *a*, and those delineated into specific H⋯H, O⋯H/H⋯O, F⋯H/H⋯F, C⋯F/⋯C and C⋯O/O⋯C contacts (McKinnon *et al.*, 2007 ▸) are illustrated in Fig. 9 ▸ *b*-*e*; the percentage contributions from the different inter-atomic contacts to the Hirshfeld surface are summarized in Table 3 ▸. The relatively small percentage contribution from H⋯H contacts to the Hirshfeld surface in the overall packing of (I) is due to the formation of a wide range of different inter­molecular inter­actions between the constituent cations, dianions and solvent ethanol mol­ecule. This is well-evidenced in the long list of contacts in Table 3 ▸. The presence of two tri­fluoro­methyl groups in each of the independent cations results in a major contribution from fluorine atoms to the Hirshfeld surface of (I), as highlighted in Table 3 ▸. Indeed, the major contributor of contacts to the surface is of the type F⋯H/H⋯F, at 41.2%. Many of these occur in the inter-layer region at separations greater than the sum of the van der Waals radii.

The presence of a cone-shaped tip at *d*_e_ + *d*_i_ 2.2 Å in the fingerprint plot delineated into H⋯H contacts in Fig. 9 ▸ *b*, is an indication of the short inter­atomic H⋯H contact between symmetry-related piperidinium-H34*A* and ethanol-H39*A* atoms, Table 2 ▸. The other short H⋯H contacts summarized in Table 2 ▸ occur between the hydrogen atoms of the cations and dianion within the asymmetric unit. In the fingerprint plot delineated into O⋯H/H⋯O contacts, Fig. 9 ▸ *c*, the pair of long spikes with their tips at *d*_e_ + *d*_i_ ∼1.8 Å are due to the O—H⋯O and N—H⋯O hydrogen bonds involving the carboxyl­ate-O4 atom of the dianion whereas the points corresponding to N—H⋯O hydrogen bonds involving the O3 and O5 atoms of the dianion and those involved in short inter­atomic O⋯H contacts, Table 2 ▸, are merged within the plot. The pair of conical tips at *d*_e_ + *d*_i_ ∼2.5 Å in the fingerprint plot delineated into F⋯H/H⋯F contacts, Fig. 9 ▸ *d*, represent the presence of these short contacts. The effect of inter­molecular C—F⋯π/π⋯F—C and short inter­atomic C⋯F/F⋯C contacts on the mol­ecular packing, Table 3 ▸, results in a small but measurable contribution of 2.8% to the Hirshfeld surface of (I) and are viewed as the pair of forceps-like tips at *d*_e_ + *d*_i_ ∼3.1 Å in Fig. 9 ▸ *e*. The presence of short inter­atomic C⋯O/O⋯C contacts involving the hydroxyl-O1 and -O2 atoms are characterized as a pair of leaf-like tips at *d*_e_ + *d*_i_ ∼3.0 Å in Fig. 9 ▸ *f*. Finally, the presence of π–π stacking inter­actions between the (C4–C9) ring of the O1-cation and the (C21–C26) and N3-pyridyl rings of the O2-cation are reflected in the 3.4 and 3.3% contributions from C⋯C contacts to the Hirshfeld surfaces of the individual cations; although the contribution from these contacts to the surfaces in the overall structure of (I) is negligible as these are embedded within the asymmetric unit.

## Database survey   

As indicated in the *Chemical context*, the specific enanti­omer of *Lariam* is important in terms of pharmacological activity. Hence, considerable investment has been made in attempting to resolve the enanti­omers by salt formation. During such studies, a seemingly high propensity towards kryptoracemic behaviour has been revealed. Kryptoracemic behaviour is related to the rare phenomenon where enanti­omeric mol­ecules crystallize in one of the 65 Sohncke space groups, *i.e*. space groups which lack an inversion centre, a rotatory inversion axis, a glide plane or a mirror plane. In these circumstances, the enanti­omeric mol­ecules are related by non-crystallographic symmetry, *e.g*. a non-crystallographic centre of inversion. A review of this phenomenon has appeared for organic compounds (Fábián & Brock, 2010 ▸) where such behaviour is found in only 0.1% of structures. There are about 30 mefloquine/derivatives in the Cambridge Structural Database (Groom *et al.*, 2016 ▸) and of these, there are two examples of kryptoracemates (Jotani *et al.*, 2016 ▸; Wardell, Wardell *et al.*, 2016 ▸). Further, in a very recent study, 34 new mefloquine salts were reported of which two were kryptoracemates (Engwerda *et al.*, 2019 ▸). Such a high adoption of kryptoracemic behaviour by these species suggest that further, systematic structural studies are warranted.

## Synthesis and crystallization   

A solution of mefloquinium chloride (1 mmol) and sodium succinate (2 mmol) in ethanol (15 ml) was refluxed for 30 min. The reaction mixture was left at room temperature and after three days, colourless platy crystals of (I) were collected; m.p. 505–505 K. Yield of recrystallized product 65%.

^1^H NMR (DMSO-*d*_6_): δ: 1.15–1.27 (2H, *m*), 1.32–1.47 (6H, *m*), 1.48–1.57 (2H, *br. d*), 1.65–1.74 (2H, *br. d*), 2.33 (4H, *s*; succinate), 2.58–2.67 (2H, *br. t*), 3.00–3.11 (4H, *m*), 5.58 (2H, *d*, *J* = 8 Hz), 7.95 (2H, *t*, *J* = 8 Hz), 8.10 (2H, *s*), 8.37 (2H, *t*, *J* = 7.2 Hz), 8.75 (2H, *d*, *J* = 8 Hz), resonances due to OH and NH were not observed. Resonances due to ethanol solvate were also present: 3.45 (*q*, *J* = 7.0 Hz) and 1.07 (*t*, *J* = 7.0 Hz). ^13^C NMR (DMSO-*d*_6_): δ: 22.92, 24.28, 24.71, 31.76, 45.55, 60.57, 70.33, 115.58, 119.89 (*J*_C,F_ = 273.8 Hz), 122.34, 122.64 (*J*_C,F_ = 271.7 Hz), 127.77 (*J*_C,F_ = 29.0 Hz), 127.76, 129.40, 129.9 (*J*_C,F_ = 5.2Hz), 142.74, 146.56 (*J*_C,F_ = 34.5 Hz), 153.13, 175.32). ^19^F NMR (DMSO-*d*_6_); δ: −58.83, −66.63. IR (cm^−1^) 3500–2100 (*br*), 1589 (*br*), 1514, 1454, 1430, 1371, 1312, 1267, 1217, 1182, 1111, 1053, 1018, 986, 941,910, 837, 777, 546, 445.

## Refinement   

Crystal data, data collection and structure refinement details are summarized in Table 4 ▸. The carbon-bound H atoms were placed in calculated positions (C—H = 0.95–1.00 Å) and were included in the refinement in the riding-model approximation, with *U*_iso_(H) set to 1.2–1.5*U*_eq_(C). The O- and N-bound H atoms were refined with distance restraints 0.84±0.01 and 0.88±0.01 Å, respectively, and refined with *U*_iso_(H) = 1.5*U*_eq_(O) and 1.2*U*_eq_(N), respectively. Owing to poor agreement, the (0
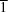
2) reflection was omitted from the final cycles of refinement.

## Supplementary Material

Crystal structure: contains datablock(s) I, global. DOI: 10.1107/S2056989019009654/hb7838sup1.cif

Structure factors: contains datablock(s) I. DOI: 10.1107/S2056989019009654/hb7838Isup2.hkl

Supporting information file. DOI: 10.1107/S2056989019009654/hb7838Isup3.cml

CCDC reference: 1938793

Additional supporting information:  crystallographic information; 3D view; checkCIF report

## Figures and Tables

**Table 1:** Hydrogen-bond geometry (Å, °) *Cg*1 is the centroid of the (N1,C1–C4,C9) ring.

*D*—H⋯*A*	*D*—H	H⋯*A*	*D*⋯*A*	*D*—H⋯*A*
N2—H1*N*⋯O1	0.88 (3)	2.49 (3)	2.806 (4)	102 (2)
N4—H3*N*⋯O2	0.88 (2)	2.54 (3)	2.863 (4)	103 (2)
O1—H1*O*⋯O6	0.84 (3)	1.81 (3)	2.653 (3)	175 (2)
O2—H2*O*⋯O4^i^	0.84 (2)	1.82 (2)	2.656 (3)	170 (3)
N2—H1*N*⋯O5^ii^	0.88 (3)	1.97 (3)	2.830 (4)	165 (3)
N2—H2*N*⋯O4^i^	0.88 (3)	1.82 (3)	2.694 (4)	173 (3)
N4—H3*N*⋯O3^ii^	0.88 (2)	1.99 (3)	2.832 (4)	161 (3)
N4—H4*N*⋯O6	0.89 (3)	1.92 (3)	2.789 (4)	168 (3)
O7—H7*O*⋯O5	0.85 (3)	1.88 (3)	2.729 (4)	176 (7)
C30—H30⋯O7	1.00	2.40	3.296 (4)	149
C10—F3⋯*Cg*1^iii^	1.32 (1)	3.28 (1)	4.101 (3)	120 (1)

Symmetry codes: (i) 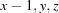; (ii) 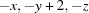; (iii) 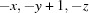.

**Table 2:** Summary of short inter­atomic contacts (Å) in (I)*^*a*^*

Contact	Distance	Symmetry operation
F1⋯H6	2.50	1 + *x*, *y*, *z*
F6⋯C32	3.096 (4)	*x*, −1 + *y*, *z*
O1⋯H37*B*	2.45	*x*, *y*, *z*
O1⋯C38	3.038 (4)	-*x*, −*y*, −1 − *z*
O2⋯H36*B*	2.50	−1 + *x*, *y*, *z*
O2⋯C36	3.090 (4)	−1 + *x*, *y*, *z*
O4⋯H12	2.53	1 + *x*, *y*, *z*
O6⋯H22	2.50	*x*, *y*, *z*
O7⋯H31*B*	2.55	*x*, *y*, *z*
H1*O*⋯H37*B*	2.14	*x*, *y*, *z*
H12⋯H29	2.06	*x*, *y*, *z*
H34*A*⋯H39*A*	2.22	-*x*, 2 − *y*, −*z*

Notes: (*a*) The inter­atomic distances are calculated in *Crystal Explorer* (Turner *et al.*, 2017 ▸) whereby the *X*—H bond lengths are adjusted to their neutron values.

**Table 3:** Percentage contributions of inter­atomic contacts to the Hirshfeld surface for (I)

Contact	Percentage contribution
H⋯H	29.8
O⋯H/H⋯O	14.0
F⋯H/H⋯F	41.2
F⋯F	5.7
C⋯H/H⋯C	4.1
C⋯F/F⋯C	2.8
N⋯H/H⋯N	1.0
C⋯N/N⋯C	0.5
C⋯O/O⋯C	0.3
O⋯O	0.2
F⋯N/N⋯F	0.2
C⋯C	0.2
F⋯O/O⋯F	0.1

**Table 4:** Experimental details

Crystal data	
Chemical formula	2C_17_H_17_F_6_N_2_O^+^·C_4_H_4_O_4_^2−^·C_2_H_6_O
*M*_r_	920.79
Crystal system, space group	Triclinic, *P* 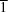
Temperature (K)	120
*a*, *b*, *c* (Å)	10.0405 (2), 14.6482 (4), 14.6547 (4)
α, β, γ (°)	100.745 (1), 93.830 (2), 98.497 (2)
*V* (Å^3^)	2084.41 (9)
*Z*	2
Radiation type	Mo *K*α
μ (mm^−1^)	0.14
Crystal size (mm)	0.42 × 0.05 × 0.03

Data collection	
Diffractometer	Bruker–Nonius Roper CCD camera on κ-goniostat
Absorption correction	Multi-scan (*SADABS*;Sheldrick, 2007 ▸)
*T*_min_, *T*_max_	0.849, 1.000
No. of measured, independent and observed [*I* > 2σ(*I*)] reflections	42885, 9543, 6505
*R*_int_	0.085
(sin θ/λ)_max_ (Å^−1^)	0.651

Refinement	
*R*[*F* ^2^ > 2σ(*F* ^2^)], *wR*(*F* ^2^), *S*	0.074, 0.180, 1.04
No. of reflections	9543
No. of parameters	590
No. of restraints	7
H-atom treatment	H atoms treated by a mixture of independent and constrained refinement
Δρ_max_, Δρ_min_ (e Å^−3^)	0.60, −0.58

Computer programs: *DENZO* (Otwinowski & Minor, 1997 ▸) and *COLLECT* (Hooft, 1998 ▸), *SHELXS97* (Sheldrick, 2008 ▸), *SHELXL2014* (Sheldrick, 2015 ▸), *ORTEP-3 for Windows* (Farrugia, 2012 ▸), *DIAMOND* (Brandenburg, 2006 ▸) and *publCIF* (Westrip, 2010 ▸).

## supplementary crystallographic information

### Crystal data

**Table:**

2C_17_H_17_F_6_N_2_O^+^·C_4_H_4_O_4_^2^^−^·C_2_H_6_O	*Z* = 2
*M**_r_* = 920.79	*F*(000) = 952
Triclinic, *P*1	*D*_x_ = 1.467 Mg m^−^^3^
*a* = 10.0405 (2) Å	Mo *K*α radiation, λ = 0.71073 Å
*b* = 14.6482 (4) Å	Cell parameters from 32862 reflections
*c* = 14.6547 (4) Å	θ = 2.9–27.5°
α = 100.745 (1)°	µ = 0.14 mm^−^^1^
β = 93.830 (2)°	*T* = 120 K
γ = 98.497 (2)°	Plate, colourless
*V* = 2084.41 (9) Å^3^	0.42 × 0.05 × 0.03 mm

### Data collection

**Table:**

Bruker–Nonius Roper CCD camera on κ-goniostat diffractometer	9543 independent reflections
Radiation source: Bruker–Nonius FR591 rotating anode	6505 reflections with *I* > 2σ(*I*)
Graphite monochromator	*R*_int_ = 0.085
Detector resolution: 9.091 pixels mm^-1^	θ_max_ = 27.6°, θ_min_ = 2.9°
φ & ω scans	*h* = −12→13
Absorption correction: multi-scan (*SADABS*;Sheldrick, 2007)	*k* = −18→19
*T*_min_ = 0.849, *T*_max_ = 1.000	*l* = −19→19
42885 measured reflections	

### Refinement

**Table:**

Refinement on *F*^2^	Primary atom site location: structure-invariant direct methods
Least-squares matrix: full	Secondary atom site location: difference Fourier map
*R*[*F*^2^ > 2σ(*F*^2^)] = 0.074	Hydrogen site location: mixed
*wR*(*F*^2^) = 0.180	H atoms treated by a mixture of independent and constrained refinement
*S* = 1.04	*w* = 1/[σ^2^(*F*_o_^2^) + (0.0525*P*)^2^ + 4.9226*P*] where *P* = (*F*_o_^2^ + 2*F*_c_^2^)/3
9543 reflections	(Δ/σ)_max_ < 0.001
590 parameters	Δρ_max_ = 0.60 e Å^−^^3^
7 restraints	Δρ_min_ = −0.58 e Å^−^^3^

### Special details

**Table:**

Geometry. All esds (except the esd in the dihedral angle between two l.s. planes) are estimated using the full covariance matrix. The cell esds are taken into account individually in the estimation of esds in distances, angles and torsion angles; correlations between esds in cell parameters are only used when they are defined by crystal symmetry. An approximate (isotropic) treatment of cell esds is used for estimating esds involving l.s. planes.
Refinement. Owing to poor agreement, possibly to interference from the beam-stop, one reflection, i.e. (0 -1 2), was omitted from the final cycles of refinement.

### Fractional atomic coordinates and isotropic or equivalent isotropic displacement parameters (Å^2^)

**Table:**

	*x*	*y*	*z*	*U*_iso_*/*U*_eq_	
F1	0.2808 (2)	0.67260 (18)	0.16134 (18)	0.0504 (7)	
F2	0.2712 (2)	0.65790 (19)	0.01402 (19)	0.0506 (7)	
F3	0.2412 (2)	0.53596 (15)	0.07419 (19)	0.0430 (6)	
F4	−0.1087 (2)	0.38548 (14)	0.16771 (16)	0.0344 (5)	
F5	−0.03229 (19)	0.48942 (14)	0.29119 (15)	0.0319 (5)	
F6	−0.2296 (2)	0.40671 (14)	0.28320 (16)	0.0364 (5)	
O1	−0.0896 (2)	0.82971 (15)	−0.04003 (16)	0.0212 (5)	
H1O	−0.061 (4)	0.869 (2)	0.0096 (16)	0.032*	
N1	−0.0028 (2)	0.57189 (18)	0.12924 (19)	0.0207 (6)	
N2	−0.3489 (3)	0.78367 (18)	−0.13680 (19)	0.0190 (5)	
H1N	−0.289 (3)	0.8273 (18)	−0.152 (2)	0.023*	
H2N	−0.390 (3)	0.811 (2)	−0.0906 (17)	0.023*	
C1	0.0642 (3)	0.6271 (2)	0.0803 (2)	0.0198 (6)	
C2	0.0089 (3)	0.6897 (2)	0.0331 (2)	0.0190 (6)	
H2	0.0631	0.7264	−0.0019	0.023*	
C3	−0.1251 (3)	0.6968 (2)	0.0384 (2)	0.0188 (6)	
C4	−0.2021 (3)	0.6417 (2)	0.0930 (2)	0.0181 (6)	
C5	−0.3393 (3)	0.6472 (2)	0.1083 (2)	0.0223 (7)	
H5	−0.3852	0.6885	0.0799	0.027*	
C6	−0.4056 (3)	0.5940 (2)	0.1630 (2)	0.0237 (7)	
H6	−0.4971	0.5989	0.1728	0.028*	
C7	−0.3403 (3)	0.5318 (2)	0.2053 (3)	0.0260 (7)	
H7	−0.3881	0.4952	0.2435	0.031*	
C8	−0.2090 (3)	0.5233 (2)	0.1922 (2)	0.0217 (7)	
C9	−0.1351 (3)	0.5793 (2)	0.1366 (2)	0.0195 (6)	
C10	0.2142 (3)	0.6225 (2)	0.0822 (2)	0.0244 (7)	
C11	−0.1435 (3)	0.4521 (2)	0.2331 (3)	0.0259 (7)	
C12	−0.1883 (3)	0.7644 (2)	−0.0125 (2)	0.0184 (6)	
H12	−0.2441	0.8000	0.0309	0.022*	
C13	−0.2802 (3)	0.7122 (2)	−0.1001 (2)	0.0176 (6)	
H13	−0.3506	0.6652	−0.0815	0.021*	
C14	−0.4440 (3)	0.7429 (2)	−0.2226 (2)	0.0263 (7)	
H14A	−0.4816	0.7940	−0.2457	0.032*	
H14B	−0.5202	0.6991	−0.2071	0.032*	
C15	−0.3715 (4)	0.6904 (3)	−0.2983 (2)	0.0295 (8)	
H15A	−0.3033	0.7359	−0.3196	0.035*	
H15B	−0.4376	0.6596	−0.3524	0.035*	
C16	−0.3015 (3)	0.6160 (2)	−0.2630 (2)	0.0262 (7)	
H16A	−0.3704	0.5665	−0.2484	0.031*	
H16B	−0.2501	0.5861	−0.3123	0.031*	
C17	−0.2054 (3)	0.6606 (2)	−0.1756 (2)	0.0201 (6)	
H17A	−0.1649	0.6110	−0.1517	0.024*	
H17B	−0.1312	0.7056	−0.1918	0.024*	
F7	−0.6032 (3)	0.6297 (2)	0.3672 (3)	0.0874 (12)	
F8	−0.6420 (2)	0.7695 (2)	0.3979 (2)	0.0572 (8)	
F9	−0.5432 (2)	0.7145 (2)	0.50201 (19)	0.0670 (9)	
F10	−0.1334 (2)	0.67918 (15)	0.55849 (15)	0.0343 (5)	
F11	−0.1959 (2)	0.55632 (14)	0.44945 (16)	0.0371 (5)	
F12	0.0147 (2)	0.60063 (14)	0.49868 (15)	0.0332 (5)	
O2	−0.4008 (2)	0.92583 (16)	0.18909 (16)	0.0236 (5)	
H2O	−0.424 (4)	0.897 (2)	0.1338 (12)	0.035*	
N3	−0.3160 (3)	0.71100 (19)	0.4089 (2)	0.0229 (6)	
N4	−0.1656 (3)	1.06682 (18)	0.20532 (19)	0.0186 (5)	
H3N	−0.240 (2)	1.071 (2)	0.172 (2)	0.022*	
H4N	−0.114 (3)	1.036 (2)	0.168 (2)	0.022*	
C18	−0.4157 (3)	0.7498 (2)	0.3795 (2)	0.0239 (7)	
C19	−0.4077 (3)	0.8153 (2)	0.3206 (2)	0.0221 (7)	
H19	−0.4848	0.8413	0.3036	0.027*	
C20	−0.2853 (3)	0.8408 (2)	0.2883 (2)	0.0188 (6)	
C21	−0.1731 (3)	0.7995 (2)	0.3149 (2)	0.0188 (6)	
C22	−0.0438 (3)	0.8172 (2)	0.2819 (2)	0.0191 (6)	
H22	−0.0287	0.8594	0.2402	0.023*	
C23	0.0591 (3)	0.7740 (2)	0.3097 (2)	0.0229 (7)	
H23	0.1448	0.7858	0.2864	0.027*	
C24	0.0399 (3)	0.7124 (2)	0.3722 (2)	0.0224 (7)	
H24	0.1126	0.6831	0.3907	0.027*	
C25	−0.0821 (3)	0.6942 (2)	0.4067 (2)	0.0223 (7)	
C26	−0.1939 (3)	0.7360 (2)	0.3771 (2)	0.0191 (6)	
C27	−0.5505 (3)	0.7155 (3)	0.4130 (3)	0.0339 (8)	
C28	−0.1002 (3)	0.6326 (2)	0.4778 (2)	0.0259 (7)	
C29	−0.2730 (3)	0.9119 (2)	0.2244 (2)	0.0182 (6)	
H29	−0.2221	0.8882	0.1710	0.022*	
C30	−0.1964 (3)	1.0076 (2)	0.2770 (2)	0.0187 (6)	
H30	−0.1091	0.9978	0.3076	0.022*	
C31	−0.0944 (3)	1.1651 (2)	0.2457 (2)	0.0243 (7)	
H31A	−0.0841	1.2015	0.1953	0.029*	
H31B	−0.0029	1.1628	0.2739	0.029*	
C32	−0.1729 (3)	1.2135 (2)	0.3186 (3)	0.0291 (8)	
H32A	−0.2611	1.2213	0.2891	0.035*	
H32B	−0.1223	1.2768	0.3467	0.035*	
C33	−0.1966 (4)	1.1559 (2)	0.3949 (3)	0.0309 (8)	
H33A	−0.1087	1.1505	0.4265	0.037*	
H33B	−0.2494	1.1880	0.4420	0.037*	
C34	−0.2739 (3)	1.0578 (2)	0.3511 (2)	0.0253 (7)	
H34A	−0.2880	1.0202	0.4002	0.030*	
H34B	−0.3638	1.0634	0.3226	0.030*	
O3	0.3721 (2)	0.87780 (16)	−0.09688 (16)	0.0245 (5)	
O4	0.5232 (2)	0.85378 (16)	0.00986 (16)	0.0246 (5)	
O5	0.1931 (2)	1.05633 (15)	0.18195 (15)	0.0217 (5)	
O6	0.0086 (2)	0.96016 (15)	0.10987 (16)	0.0215 (5)	
C35	0.4142 (3)	0.8829 (2)	−0.0146 (2)	0.0194 (6)	
C36	0.3374 (3)	0.9243 (2)	0.0649 (2)	0.0212 (6)	
H36A	0.3096	0.8752	0.1011	0.025*	
H36B	0.3993	0.9762	0.1072	0.025*	
C37	0.2127 (3)	0.9620 (2)	0.0338 (2)	0.0188 (6)	
H37A	0.2407	1.0150	0.0024	0.023*	
H37B	0.1535	0.9118	−0.0123	0.023*	
C38	0.1328 (3)	0.9955 (2)	0.1142 (2)	0.0176 (6)	
O7	0.1253 (3)	1.0539 (3)	0.3588 (2)	0.0587 (9)	
H7O	0.147 (6)	1.052 (4)	0.3036 (17)	0.088*	
C39	0.2349 (6)	1.0663 (5)	0.4209 (4)	0.083 (2)	
H39A	0.2025	1.0473	0.4780	0.100*	
H39B	0.2682	1.1349	0.4375	0.100*	
C40	0.3465 (6)	1.0231 (7)	0.4013 (4)	0.105 (3)	
H40A	0.3264	0.9569	0.4070	0.158*	
H40B	0.4242	1.0552	0.4456	0.158*	
H40C	0.3679	1.0265	0.3377	0.158*	

### Atomic displacement parameters (Å^2^)

**Table:**

	*U*^11^	*U*^22^	*U*^33^	*U*^12^	*U*^13^	*U*^23^
F1	0.0210 (11)	0.0686 (16)	0.0492 (15)	0.0145 (10)	−0.0092 (10)	−0.0207 (13)
F2	0.0212 (11)	0.0813 (18)	0.0645 (17)	0.0170 (11)	0.0168 (10)	0.0419 (15)
F3	0.0250 (11)	0.0294 (11)	0.0778 (18)	0.0147 (9)	0.0100 (11)	0.0087 (11)
F4	0.0393 (12)	0.0239 (10)	0.0413 (13)	0.0132 (8)	0.0039 (9)	0.0037 (9)
F5	0.0298 (11)	0.0327 (11)	0.0343 (12)	0.0066 (8)	−0.0040 (9)	0.0112 (9)
F6	0.0360 (11)	0.0318 (11)	0.0482 (14)	0.0061 (9)	0.0097 (10)	0.0225 (10)
O1	0.0189 (11)	0.0214 (11)	0.0216 (13)	0.0002 (8)	−0.0014 (9)	0.0033 (9)
N1	0.0161 (12)	0.0221 (13)	0.0230 (15)	0.0049 (10)	−0.0009 (10)	0.0016 (11)
N2	0.0162 (13)	0.0197 (13)	0.0201 (15)	0.0033 (10)	−0.0002 (10)	0.0017 (11)
C1	0.0158 (14)	0.0208 (15)	0.0208 (17)	0.0049 (11)	−0.0011 (12)	−0.0011 (13)
C2	0.0147 (14)	0.0213 (15)	0.0200 (17)	0.0024 (11)	0.0014 (11)	0.0027 (13)
C3	0.0202 (15)	0.0163 (14)	0.0193 (17)	0.0047 (11)	−0.0001 (12)	0.0017 (12)
C4	0.0139 (14)	0.0192 (15)	0.0195 (17)	0.0027 (11)	−0.0001 (11)	0.0002 (12)
C5	0.0183 (15)	0.0226 (16)	0.0259 (18)	0.0063 (12)	0.0009 (12)	0.0025 (14)
C6	0.0125 (14)	0.0267 (16)	0.0312 (19)	0.0030 (12)	0.0026 (12)	0.0043 (14)
C7	0.0192 (16)	0.0276 (17)	0.032 (2)	0.0017 (13)	0.0030 (13)	0.0078 (15)
C8	0.0211 (15)	0.0187 (15)	0.0241 (18)	0.0036 (12)	−0.0007 (12)	0.0021 (13)
C9	0.0189 (15)	0.0173 (14)	0.0216 (17)	0.0049 (11)	0.0002 (12)	0.0010 (12)
C10	0.0178 (15)	0.0251 (16)	0.0297 (19)	0.0058 (12)	−0.0008 (13)	0.0036 (14)
C11	0.0230 (16)	0.0239 (16)	0.031 (2)	0.0034 (13)	0.0022 (14)	0.0075 (15)
C12	0.0127 (14)	0.0198 (15)	0.0229 (17)	0.0040 (11)	0.0000 (11)	0.0047 (13)
C13	0.0156 (14)	0.0219 (15)	0.0165 (16)	0.0045 (11)	0.0022 (11)	0.0052 (12)
C14	0.0243 (16)	0.0326 (18)	0.0203 (18)	0.0088 (13)	−0.0045 (13)	0.0002 (14)
C15	0.0336 (19)	0.0365 (19)	0.0172 (18)	0.0116 (15)	−0.0039 (14)	0.0003 (15)
C16	0.0264 (17)	0.0268 (17)	0.0230 (19)	0.0041 (13)	0.0033 (13)	−0.0013 (14)
C17	0.0205 (15)	0.0176 (15)	0.0228 (18)	0.0039 (11)	0.0038 (12)	0.0045 (13)
F7	0.0547 (18)	0.0618 (19)	0.127 (3)	−0.0282 (14)	0.0462 (19)	−0.0129 (19)
F8	0.0204 (11)	0.098 (2)	0.0746 (19)	0.0233 (12)	0.0199 (11)	0.0562 (17)
F9	0.0271 (12)	0.144 (3)	0.0493 (17)	0.0202 (14)	0.0152 (11)	0.0595 (19)
F10	0.0382 (12)	0.0429 (12)	0.0272 (12)	0.0135 (9)	0.0061 (9)	0.0139 (10)
F11	0.0348 (12)	0.0283 (11)	0.0494 (14)	−0.0020 (9)	0.0005 (10)	0.0177 (10)
F12	0.0325 (11)	0.0342 (11)	0.0385 (13)	0.0146 (9)	−0.0008 (9)	0.0159 (10)
O2	0.0196 (11)	0.0317 (13)	0.0199 (13)	0.0086 (9)	−0.0029 (9)	0.0047 (10)
N3	0.0180 (13)	0.0271 (14)	0.0257 (16)	0.0047 (10)	0.0039 (11)	0.0090 (12)
N4	0.0184 (13)	0.0221 (13)	0.0167 (14)	0.0072 (10)	0.0028 (10)	0.0046 (11)
C18	0.0174 (15)	0.0301 (17)	0.0241 (18)	0.0027 (12)	0.0015 (12)	0.0058 (14)
C19	0.0144 (14)	0.0258 (16)	0.0272 (19)	0.0059 (12)	0.0034 (12)	0.0053 (14)
C20	0.0171 (14)	0.0190 (15)	0.0186 (17)	0.0019 (11)	−0.0005 (11)	0.0006 (12)
C21	0.0143 (14)	0.0207 (15)	0.0198 (17)	0.0015 (11)	−0.0004 (11)	0.0021 (13)
C22	0.0184 (15)	0.0215 (15)	0.0172 (16)	0.0033 (11)	0.0008 (11)	0.0034 (12)
C23	0.0138 (14)	0.0256 (16)	0.0281 (19)	0.0037 (12)	0.0014 (12)	0.0024 (14)
C24	0.0211 (15)	0.0201 (15)	0.0252 (18)	0.0057 (12)	−0.0020 (12)	0.0023 (13)
C25	0.0219 (15)	0.0207 (15)	0.0232 (18)	0.0026 (12)	−0.0024 (12)	0.0041 (13)
C26	0.0187 (15)	0.0184 (14)	0.0185 (17)	0.0024 (11)	−0.0009 (12)	0.0007 (12)
C27	0.0206 (17)	0.044 (2)	0.039 (2)	0.0029 (15)	0.0051 (15)	0.0150 (18)
C28	0.0248 (17)	0.0272 (17)	0.0271 (19)	0.0069 (13)	0.0003 (13)	0.0079 (15)
C29	0.0141 (14)	0.0211 (15)	0.0196 (17)	0.0051 (11)	0.0009 (11)	0.0030 (13)
C30	0.0179 (14)	0.0226 (15)	0.0160 (16)	0.0057 (11)	0.0026 (11)	0.0026 (12)
C31	0.0212 (16)	0.0232 (16)	0.0284 (19)	0.0010 (12)	−0.0007 (13)	0.0080 (14)
C32	0.0265 (17)	0.0225 (16)	0.036 (2)	0.0081 (13)	−0.0040 (14)	−0.0009 (15)
C33	0.040 (2)	0.0285 (18)	0.0228 (19)	0.0104 (15)	0.0041 (15)	−0.0023 (15)
C34	0.0284 (17)	0.0270 (17)	0.0219 (18)	0.0071 (13)	0.0099 (13)	0.0041 (14)
O3	0.0218 (11)	0.0347 (13)	0.0185 (13)	0.0113 (9)	0.0004 (9)	0.0046 (10)
O4	0.0181 (11)	0.0343 (13)	0.0224 (13)	0.0115 (9)	−0.0002 (9)	0.0033 (10)
O5	0.0206 (11)	0.0249 (11)	0.0186 (12)	0.0031 (9)	0.0006 (9)	0.0023 (9)
O6	0.0150 (10)	0.0225 (11)	0.0261 (13)	0.0024 (8)	0.0035 (9)	0.0027 (9)
C35	0.0149 (14)	0.0185 (15)	0.0261 (18)	0.0035 (11)	0.0024 (12)	0.0071 (13)
C36	0.0194 (15)	0.0266 (16)	0.0194 (17)	0.0082 (12)	0.0030 (12)	0.0056 (13)
C37	0.0146 (14)	0.0222 (15)	0.0193 (17)	0.0042 (11)	0.0012 (11)	0.0028 (13)
C38	0.0170 (14)	0.0190 (15)	0.0189 (17)	0.0068 (11)	0.0015 (11)	0.0061 (12)
O7	0.0374 (16)	0.111 (3)	0.0352 (18)	0.0244 (17)	0.0060 (13)	0.0237 (19)
C39	0.074 (4)	0.136 (6)	0.040 (3)	0.053 (4)	−0.015 (3)	−0.004 (3)
C40	0.052 (3)	0.220 (9)	0.058 (4)	0.060 (4)	0.004 (3)	0.036 (5)

### Geometric parameters (Å, º)

**Table:**

F1—C10	1.328 (4)	N4—C31	1.502 (4)
F2—C10	1.333 (4)	N4—C30	1.502 (4)
F3—C10	1.321 (4)	N4—H3N	0.882 (10)
F4—C11	1.338 (4)	N4—H4N	0.882 (10)
F5—C11	1.338 (4)	C18—C19	1.403 (5)
F6—C11	1.352 (4)	C18—C27	1.518 (5)
O1—C12	1.406 (4)	C19—C20	1.373 (4)
O1—H1O	0.840 (10)	C19—H19	0.9500
N1—C1	1.319 (4)	C20—C21	1.419 (4)
N1—C9	1.358 (4)	C20—C29	1.522 (4)
N2—C13	1.495 (4)	C21—C22	1.421 (4)
N2—C14	1.497 (4)	C21—C26	1.423 (4)
N2—H1N	0.880 (10)	C22—C23	1.366 (4)
N2—H2N	0.883 (10)	C22—H22	0.9500
C1—C2	1.401 (4)	C23—C24	1.404 (5)
C1—C10	1.517 (4)	C23—H23	0.9500
C2—C3	1.370 (4)	C24—C25	1.365 (5)
C2—H2	0.9500	C24—H24	0.9500
C3—C4	1.425 (4)	C25—C26	1.435 (4)
C3—C12	1.530 (4)	C25—C28	1.504 (5)
C4—C5	1.422 (4)	C29—C30	1.533 (4)
C4—C9	1.428 (4)	C29—H29	1.0000
C5—C6	1.357 (5)	C30—C34	1.519 (4)
C5—H5	0.9500	C30—H30	1.0000
C6—C7	1.406 (5)	C31—C32	1.504 (5)
C6—H6	0.9500	C31—H31A	0.9900
C7—C8	1.365 (4)	C31—H31B	0.9900
C7—H7	0.9500	C32—C33	1.532 (5)
C8—C9	1.427 (4)	C32—H32A	0.9900
C8—C11	1.503 (4)	C32—H32B	0.9900
C12—C13	1.533 (4)	C33—C34	1.529 (5)
C12—H12	1.0000	C33—H33A	0.9900
C13—C17	1.525 (4)	C33—H33B	0.9900
C13—H13	1.0000	C34—H34A	0.9900
C14—C15	1.519 (5)	C34—H34B	0.9900
C14—H14A	0.9900	O3—C35	1.236 (4)
C14—H14B	0.9900	O4—C35	1.285 (4)
C15—C16	1.529 (5)	O5—C38	1.255 (4)
C15—H15A	0.9900	O6—C38	1.271 (4)
C15—H15B	0.9900	C35—C36	1.522 (4)
C16—C17	1.527 (5)	C36—C37	1.517 (4)
C16—H16A	0.9900	C36—H36A	0.9900
C16—H16B	0.9900	C36—H36B	0.9900
C17—H17A	0.9900	C37—C38	1.517 (4)
C17—H17B	0.9900	C37—H37A	0.9900
F7—C27	1.323 (5)	C37—H37B	0.9900
F8—C27	1.331 (4)	O7—C39	1.347 (6)
F9—C27	1.304 (5)	O7—H7O	0.847 (10)
F10—C28	1.340 (4)	C39—C40	1.388 (8)
F11—C28	1.341 (4)	C39—H39A	0.9900
F12—C28	1.346 (4)	C39—H39B	0.9900
O2—C29	1.409 (3)	C40—H40A	0.9800
O2—H2O	0.841 (10)	C40—H40B	0.9800
N3—C18	1.309 (4)	C40—H40C	0.9800
N3—C26	1.363 (4)		
			
C12—O1—H1O	104 (3)	C21—C20—C29	121.6 (3)
C1—N1—C9	116.7 (3)	C22—C21—C20	123.7 (3)
C13—N2—C14	113.4 (2)	C22—C21—C26	119.0 (3)
C13—N2—H1N	111 (2)	C20—C21—C26	117.3 (3)
C14—N2—H1N	106 (2)	C23—C22—C21	120.4 (3)
C13—N2—H2N	106 (2)	C23—C22—H22	119.8
C14—N2—H2N	112 (2)	C21—C22—H22	119.8
H1N—N2—H2N	108 (3)	C22—C23—C24	120.8 (3)
N1—C1—C2	125.4 (3)	C22—C23—H23	119.6
N1—C1—C10	114.3 (3)	C24—C23—H23	119.6
C2—C1—C10	120.2 (3)	C25—C24—C23	120.8 (3)
C3—C2—C1	118.6 (3)	C25—C24—H24	119.6
C3—C2—H2	120.7	C23—C24—H24	119.6
C1—C2—H2	120.7	C24—C25—C26	120.1 (3)
C2—C3—C4	118.9 (3)	C24—C25—C28	120.7 (3)
C2—C3—C12	119.8 (3)	C26—C25—C28	119.2 (3)
C4—C3—C12	121.3 (3)	N3—C26—C21	123.3 (3)
C5—C4—C3	124.0 (3)	N3—C26—C25	117.9 (3)
C5—C4—C9	118.7 (3)	C21—C26—C25	118.8 (3)
C3—C4—C9	117.3 (3)	F9—C27—F7	107.9 (3)
C6—C5—C4	120.8 (3)	F9—C27—F8	106.4 (3)
C6—C5—H5	119.6	F7—C27—F8	105.4 (3)
C4—C5—H5	119.6	F9—C27—C18	113.5 (3)
C5—C6—C7	120.7 (3)	F7—C27—C18	111.5 (3)
C5—C6—H6	119.7	F8—C27—C18	111.6 (3)
C7—C6—H6	119.7	F10—C28—F11	107.0 (3)
C8—C7—C6	120.8 (3)	F10—C28—F12	106.1 (3)
C8—C7—H7	119.6	F11—C28—F12	106.4 (3)
C6—C7—H7	119.6	F10—C28—C25	112.0 (3)
C7—C8—C9	120.1 (3)	F11—C28—C25	113.4 (3)
C7—C8—C11	120.1 (3)	F12—C28—C25	111.4 (3)
C9—C8—C11	119.7 (3)	O2—C29—C20	111.7 (2)
N1—C9—C8	118.1 (3)	O2—C29—C30	107.7 (2)
N1—C9—C4	123.0 (3)	C20—C29—C30	110.8 (3)
C8—C9—C4	118.8 (3)	O2—C29—H29	108.9
F3—C10—F1	107.1 (3)	C20—C29—H29	108.9
F3—C10—F2	106.5 (3)	C30—C29—H29	108.9
F1—C10—F2	105.9 (3)	N4—C30—C34	110.2 (2)
F3—C10—C1	113.2 (3)	N4—C30—C29	106.9 (2)
F1—C10—C1	111.1 (3)	C34—C30—C29	113.9 (3)
F2—C10—C1	112.5 (3)	N4—C30—H30	108.6
F5—C11—F4	107.0 (3)	C34—C30—H30	108.6
F5—C11—F6	106.0 (3)	C29—C30—H30	108.6
F4—C11—F6	106.2 (3)	N4—C31—C32	110.6 (3)
F5—C11—C8	114.0 (3)	N4—C31—H31A	109.5
F4—C11—C8	112.6 (3)	C32—C31—H31A	109.5
F6—C11—C8	110.6 (3)	N4—C31—H31B	109.5
O1—C12—C3	112.0 (2)	C32—C31—H31B	109.5
O1—C12—C13	107.7 (2)	H31A—C31—H31B	108.1
C3—C12—C13	112.0 (2)	C31—C32—C33	110.5 (3)
O1—C12—H12	108.4	C31—C32—H32A	109.5
C3—C12—H12	108.4	C33—C32—H32A	109.5
C13—C12—H12	108.4	C31—C32—H32B	109.5
N2—C13—C17	110.2 (2)	C33—C32—H32B	109.5
N2—C13—C12	107.1 (2)	H32A—C32—H32B	108.1
C17—C13—C12	113.9 (2)	C34—C33—C32	109.4 (3)
N2—C13—H13	108.5	C34—C33—H33A	109.8
C17—C13—H13	108.5	C32—C33—H33A	109.8
C12—C13—H13	108.5	C34—C33—H33B	109.8
N2—C14—C15	110.6 (3)	C32—C33—H33B	109.8
N2—C14—H14A	109.5	H33A—C33—H33B	108.2
C15—C14—H14A	109.5	C30—C34—C33	110.9 (3)
N2—C14—H14B	109.5	C30—C34—H34A	109.5
C15—C14—H14B	109.5	C33—C34—H34A	109.5
H14A—C14—H14B	108.1	C30—C34—H34B	109.5
C14—C15—C16	111.4 (3)	C33—C34—H34B	109.5
C14—C15—H15A	109.4	H34A—C34—H34B	108.0
C16—C15—H15A	109.4	O3—C35—O4	123.2 (3)
C14—C15—H15B	109.4	O3—C35—C36	121.1 (3)
C16—C15—H15B	109.4	O4—C35—C36	115.7 (3)
H15A—C15—H15B	108.0	C37—C36—C35	114.3 (3)
C17—C16—C15	110.5 (3)	C37—C36—H36A	108.7
C17—C16—H16A	109.5	C35—C36—H36A	108.7
C15—C16—H16A	109.5	C37—C36—H36B	108.7
C17—C16—H16B	109.5	C35—C36—H36B	108.7
C15—C16—H16B	109.5	H36A—C36—H36B	107.6
H16A—C16—H16B	108.1	C38—C37—C36	112.7 (3)
C13—C17—C16	110.9 (2)	C38—C37—H37A	109.1
C13—C17—H17A	109.5	C36—C37—H37A	109.1
C16—C17—H17A	109.5	C38—C37—H37B	109.1
C13—C17—H17B	109.5	C36—C37—H37B	109.1
C16—C17—H17B	109.5	H37A—C37—H37B	107.8
H17A—C17—H17B	108.1	O5—C38—O6	123.4 (3)
C29—O2—H2O	113 (3)	O5—C38—C37	118.3 (3)
C18—N3—C26	116.1 (3)	O6—C38—C37	118.3 (3)
C31—N4—C30	114.0 (2)	C39—O7—H7O	112 (4)
C31—N4—H3N	108 (2)	O7—C39—C40	122.4 (5)
C30—N4—H3N	111 (2)	O7—C39—H39A	106.7
C31—N4—H4N	111 (2)	C40—C39—H39A	106.7
C30—N4—H4N	105 (2)	O7—C39—H39B	106.7
H3N—N4—H4N	108 (3)	C40—C39—H39B	106.7
N3—C18—C19	126.2 (3)	H39A—C39—H39B	106.6
N3—C18—C27	113.7 (3)	C39—C40—H40A	109.5
C19—C18—C27	120.0 (3)	C39—C40—H40B	109.5
C20—C19—C18	118.0 (3)	H40A—C40—H40B	109.5
C20—C19—H19	121.0	C39—C40—H40C	109.5
C18—C19—H19	121.0	H40A—C40—H40C	109.5
C19—C20—C21	119.0 (3)	H40B—C40—H40C	109.5
C19—C20—C29	119.4 (3)		
			
C9—N1—C1—C2	−2.4 (5)	C27—C18—C19—C20	−176.8 (3)
C9—N1—C1—C10	174.4 (3)	C18—C19—C20—C21	0.5 (5)
N1—C1—C2—C3	1.3 (5)	C18—C19—C20—C29	−179.9 (3)
C10—C1—C2—C3	−175.4 (3)	C19—C20—C21—C22	176.8 (3)
C1—C2—C3—C4	1.1 (4)	C29—C20—C21—C22	−2.9 (5)
C1—C2—C3—C12	−179.9 (3)	C19—C20—C21—C26	−2.2 (4)
C2—C3—C4—C5	176.1 (3)	C29—C20—C21—C26	178.1 (3)
C12—C3—C4—C5	−2.8 (5)	C20—C21—C22—C23	−179.1 (3)
C2—C3—C4—C9	−2.1 (4)	C26—C21—C22—C23	−0.2 (5)
C12—C3—C4—C9	178.9 (3)	C21—C22—C23—C24	−0.9 (5)
C3—C4—C5—C6	−178.4 (3)	C22—C23—C24—C25	0.1 (5)
C9—C4—C5—C6	−0.2 (5)	C23—C24—C25—C26	1.8 (5)
C4—C5—C6—C7	−0.4 (5)	C23—C24—C25—C28	−176.5 (3)
C5—C6—C7—C8	−0.1 (5)	C18—N3—C26—C21	−0.7 (5)
C6—C7—C8—C9	1.3 (5)	C18—N3—C26—C25	−179.2 (3)
C6—C7—C8—C11	−176.2 (3)	C22—C21—C26—N3	−176.6 (3)
C1—N1—C9—C8	−177.5 (3)	C20—C21—C26—N3	2.4 (5)
C1—N1—C9—C4	1.2 (5)	C22—C21—C26—C25	1.9 (4)
C7—C8—C9—N1	176.8 (3)	C20—C21—C26—C25	−179.0 (3)
C11—C8—C9—N1	−5.6 (5)	C24—C25—C26—N3	175.9 (3)
C7—C8—C9—C4	−2.0 (5)	C28—C25—C26—N3	−5.8 (4)
C11—C8—C9—C4	175.6 (3)	C24—C25—C26—C21	−2.7 (5)
C5—C4—C9—N1	−177.3 (3)	C28—C25—C26—C21	175.5 (3)
C3—C4—C9—N1	1.0 (5)	N3—C18—C27—F9	47.0 (5)
C5—C4—C9—C8	1.4 (4)	C19—C18—C27—F9	−134.4 (4)
C3—C4—C9—C8	179.7 (3)	N3—C18—C27—F7	−75.0 (4)
N1—C1—C10—F3	42.1 (4)	C19—C18—C27—F7	103.5 (4)
C2—C1—C10—F3	−140.9 (3)	N3—C18—C27—F8	167.3 (3)
N1—C1—C10—F1	−78.4 (4)	C19—C18—C27—F8	−14.2 (5)
C2—C1—C10—F1	98.6 (4)	C24—C25—C28—F10	118.1 (3)
N1—C1—C10—F2	163.0 (3)	C26—C25—C28—F10	−60.2 (4)
C2—C1—C10—F2	−20.0 (4)	C24—C25—C28—F11	−120.7 (3)
C7—C8—C11—F5	−121.8 (3)	C26—C25—C28—F11	61.1 (4)
C9—C8—C11—F5	60.6 (4)	C24—C25—C28—F12	−0.6 (4)
C7—C8—C11—F4	116.1 (3)	C26—C25—C28—F12	−178.9 (3)
C9—C8—C11—F4	−61.5 (4)	C19—C20—C29—O2	−14.1 (4)
C7—C8—C11—F6	−2.5 (5)	C21—C20—C29—O2	165.5 (3)
C9—C8—C11—F6	179.9 (3)	C19—C20—C29—C30	105.9 (3)
C2—C3—C12—O1	−15.4 (4)	C21—C20—C29—C30	−74.4 (4)
C4—C3—C12—O1	163.5 (3)	C31—N4—C30—C34	53.7 (3)
C2—C3—C12—C13	105.7 (3)	C31—N4—C30—C29	177.9 (2)
C4—C3—C12—C13	−75.4 (4)	O2—C29—C30—N4	−68.4 (3)
C14—N2—C13—C17	56.2 (3)	C20—C29—C30—N4	169.2 (2)
C14—N2—C13—C12	−179.4 (2)	O2—C29—C30—C34	53.6 (3)
O1—C12—C13—N2	−63.4 (3)	C20—C29—C30—C34	−68.9 (3)
C3—C12—C13—N2	173.1 (2)	C30—N4—C31—C32	−54.6 (3)
O1—C12—C13—C17	58.7 (3)	N4—C31—C32—C33	56.3 (4)
C3—C12—C13—C17	−64.8 (3)	C31—C32—C33—C34	−58.7 (4)
C13—N2—C14—C15	−55.5 (4)	N4—C30—C34—C33	−55.2 (4)
N2—C14—C15—C16	54.4 (4)	C29—C30—C34—C33	−175.3 (3)
C14—C15—C16—C17	−55.4 (4)	C32—C33—C34—C30	58.3 (4)
N2—C13—C17—C16	−55.8 (3)	O3—C35—C36—C37	−3.0 (4)
C12—C13—C17—C16	−176.1 (3)	O4—C35—C36—C37	177.4 (3)
C15—C16—C17—C13	56.0 (3)	C35—C36—C37—C38	175.4 (3)
C26—N3—C18—C19	−1.4 (5)	C36—C37—C38—O5	55.5 (4)
C26—N3—C18—C27	177.0 (3)	C36—C37—C38—O6	−124.1 (3)
N3—C18—C19—C20	1.5 (5)		

### Hydrogen-bond geometry (Å, º)

*Cg*1 is the centroid of the (N1,C1–C4,C9) ring.

**Table:**

*D*—H···*A*	*D*—H	H···*A*	*D*···*A*	*D*—H···*A*
N2—H1*N*···O1	0.88 (3)	2.49 (3)	2.806 (4)	102 (2)
N4—H3*N*···O2	0.88 (2)	2.54 (3)	2.863 (4)	103 (2)
O1—H1*O*···O6	0.84 (3)	1.81 (3)	2.653 (3)	175 (2)
O2—H2*O*···O4^i^	0.84 (2)	1.82 (2)	2.656 (3)	170 (3)
N2—H1*N*···O5^ii^	0.88 (3)	1.97 (3)	2.830 (4)	165 (3)
N2—H2*N*···O4^i^	0.88 (3)	1.82 (3)	2.694 (4)	173 (3)
N4—H3*N*···O3^ii^	0.88 (2)	1.99 (3)	2.832 (4)	161 (3)
N4—H4*N*···O6	0.89 (3)	1.92 (3)	2.789 (4)	168 (3)
O7—H7*O*···O5	0.85 (3)	1.88 (3)	2.729 (4)	176 (7)
C30—H30···O7	1.00	2.40	3.296 (4)	149
C10—F3···*Cg*1^iii^	1.32 (1)	3.28 (1)	4.101 (3)	120 (1)

Symmetry codes: (i) *x*−1, *y*, *z*; (ii) −*x*, −*y*+2, −*z*; (iii) −*x*, −*y*+1, −*z*.
